# The Genetic Signature of Sex-Biased Migration in Patrilocal Chimpanzees and Humans

**DOI:** 10.1371/journal.pone.0000973

**Published:** 2007-10-03

**Authors:** Kevin E. Langergraber, Heike Siedel, John C. Mitani, Richard W. Wrangham, Vernon Reynolds, Kevin Hunt, Linda Vigilant

**Affiliations:** 1 Department of Anthropology, University of Michigan, Ann Arbor, Michigan, United States of America; 2 Primatology Department, Max Planck Institute for Evolutionary Anthropology, Leipzig, Germany; 3 Department of Anthropology, Harvard University, Cambridge, Massachusetts, United States of America; 4 School of Anthropology, Oxford University, Oxford, United Kingdom; 5 Anthropology Department, Indiana University, Bloomington, Indiana, United States of America; Santa Fe Institute, United States of America

## Abstract

A large body of theoretical work suggests that analyses of variation at the maternally inherited mitochondrial (mt)DNA and the paternally inherited non-recombining portion of the Y chromosome (NRY) are a potentially powerful way to reveal the differing migratory histories of men and women across human societies. However, the few empirical studies comparing mtDNA and NRY variation and known patterns of sex-biased migration have produced conflicting results. Here we review some methodological reasons for these inconsistencies, and take them into account to provide an unbiased characterization of mtDNA and NRY variation in chimpanzees, one of the few mammalian taxa where males routinely remain in and females typically disperse from their natal groups. We show that patterns of mtDNA and NRY variation are more strongly contrasting in patrilocal chimpanzees compared with patrilocal human societies. The chimpanzee data we present here thus provide a valuable comparative benchmark of the patterns of mtDNA and NRY variation to be expected in a society with extremely female-biased dispersal.

## Introduction

Human societies show significant variation in post-marital residence practices. About 70% of human societies practice some form of patrilocality, with men remaining in and women migrating from their natal household, clan, lineage, village, or other cultural unit subsumed within a larger group of people sharing a common culture and language, often termed a `tribé in traditional societies [1], [2]. Other societies display matrilocal or bilocal migration patterns, with men and members of both sexes, respectively, leaving their birthplace to live with their mate elsewhere in the tribe [2]. In patrilocal societies, variation within tribes should be higher for the mtDNA than for the NRY, while genetic differentiation between tribes should be higher for the NRY than the mtDNA. Matrilocal societies should show the opposite patterns, while there should be no differences between mtDNA and NRY variation in bilocal tribes.

One study of Hill tribes of northern Thailand showed the predicted differences between patrilocal and matrilocal tribes in patterns of mtDNA and NRY variation [3]. However, expected patterns of mtDNA diversity were not found in a recent comparison of Central Asian patrilocal pastoral populations, where men acquire brides from outside their clan or lineage, and bilocal farmer populations, where both men and women choose brides from outside their nuclear or extended families [4]. In contrast, predicted patterns of NRY diversity were found in these same populations [4]. A third study reported no differences in patterns of mtDNA and NRY variation between patrilocal and matrilocal tribes and castes in India [5].

At least three factors may contribute to the discrepant findings of prior research comparing contemporary patterns of genetic variation and post-marital residence practices. First, analyses of mtDNA and NRY variation have been performed at a broader scale of social organization than that at which sex-biased migration actually takes place. For example, the different patterns of mtDNA and NRY diversity shown by matrilocal and patrilocal tribes in Thailand versus India may occur because only in Thailand do members of the migrating sex sometimes move out of their own tribe to join another. Migration outside the tribe is actually a rare event in traditional societies, as different tribes can have very different languages and cultures [2]. By conducting their analyses at the level of tribes within a larger tribal group, rather than at the level of the household, clan, lineage or village within a tribe, these studies did not directly examine the effects of sex-biased migration on mtDNA and NRY variation. That local processes of sex-biased migration will not necessarily affect analyses of genetic variation at broader spatial scales is shown by the finding that despite the prevalence of patrilocality across human societies [1], genetic differentiation among continents is not higher for the NRY than the mtDNA [6].

A second factor complicating attempts to compare patterns of mtDNA and NRY variation is that estimates of genetic differentiation between populations are sensitive to variables that reduce the level of within-group variation, with decreased variation resulting in larger genetic differentiation. For example, an early study of global genetic variation found that genetic differentiation among continents was larger for the NRY than the mtDNA [7], apparently in part because the levels of diversity were higher for the mtDNA than for the NRY. In contrast, recent research utilizing NRY markers that were as polymorphic as those employed for the mtDNA resulted in equal NRY and mtDNA genetic differentiation [6]. The issue of relative marker variability applies not only when comparing genetic differentiation between NRY and mtDNA, but also when comparing genetic differentiation across studies that have used different methods to assay variation for a given genetic system. For example, while the study of matrilocal Thai Hill tribes [3] typed nine microsatellite markers to assay NRY variation, the study of matrilocal Indian tribes [5] used only six, which may have led to lower estimates of NRY variation within tribes and correspondingly larger NRY genetic differentiation between tribes. Sex differences in effective population size can also contribute to lower within-group NRY variation compared with that shown by mtDNA. Because polygyny and male-biased adult mortality characterize many human societies, the effective population size of men is generally thought to be smaller than that of women [8].

Finally, a third possible reason for the contradictory findings of prior research is that we do not know for how long and how consistently each of the sampled societies has practiced their particular form of post-marital residence. One of the societies in the Central Asian study has practiced its contemporary form of post-marital residence for several centuries [4]. For the Thai and Indian populations and for most human societies, however, the evolutionary history of sex-biased migration is only poorly understood and is likely to be complex. The switch from a foraging to a food-producing lifestyle, which began ∼10,000 years ago in some populations but much more recently in others, was likely accompanied by a shift from bilocality to patrilocality [9]. The ethnographic record also provides many examples of societies undergoing recent and rapid changes in their post-marital residence patterns [9]. In such cases, there may have been insufficient time for the opposing forces of migration and genetic drift to have reached equilibrium, and levels of genetic differentiation will reflect historical rather than current conditions [10].

In contrast to the complex situation in human populations, patterns of sex-biased migration in animals are often well known from long-term behavioral observations. The currently available genetic studies of animal populations, however, furnish limited insights into how sex differences in migration affect mtDNA and NRY variation. Long-term behavioral observations indicate that migration out of bonobo groups is extremely female-biased [11]. In contrast, hamadryas baboons live in a hierarchical social system, with one male units, bands, clans and troops, and it is unclear at which level of the hierarchy, if any, migration is actually sex-biased [12]. In the bonobo and hamadryas baboon studies, samples were primarily collected from unhabituated and unidentified individuals of uncertain group membership, and analyses of mtDNA and NRY variation were conducted at the broad geographic scale of the region or population [13], [14]. While both taxa show patterns of NRY and mtDNA variation that are consistent with patrilocality, these studies do not provide a direct link between well-characterized migration histories and contemporary patterns of genetic variation. In sum, no study of humans or animals has provided an unbiased survey of mtDNA and NRY variation where both the unit and evolutionary history of sex-biased migration are known.

Here we examine patterns of mtDNA and NRY variation in four groups of wild chimpanzees located in Uganda and separated by 10–165 km. Chimpanzees live in communities consisting of 20–150 individuals, and as a result of over 180 total years of human observation at multiple field sites, their migration patterns are well understood [15]. Males are extremely hostile towards males from other communities, and male migration between communities rarely, if ever, occurs [15]. In contrast, 50–90% of females migrate from their natal community at sexual maturity to reproduce in another community [15]. Because the bonobo, the sister species of chimpanzees, also displays female-biased migration, we can infer that this migration pattern has been present for at least the 1 million years since these taxa diverged [16].

We compare the patterns of variation in chimpanzees with those inferred from published data on patrilocal human tribes belonging to five different larger cultural/geographic regions, hereafter termed ‘tribal groups’ (N = 20 tribes, Range = 2–5 tribes per tribal group) [3], [5], [17] . In addition to traditional F_ST_ based genetic differentiation estimates, we use the recently developed standardized measure of genetic differentiation [18], [19]. This measure expresses genetic differentiation as the maximum amount of genetic differentiation possible given the amount of within-group variation. It thus allows for meaningful comparisons of genetic differentiation when the amount of within-group variation is different for mtDNA and NRY, which can result from sex, group or species differences in effective population size and in the method used to assay variation, i.e., the number and mutation rate of markers [18], [19].

## Results and Discussion

**Figure 1:**
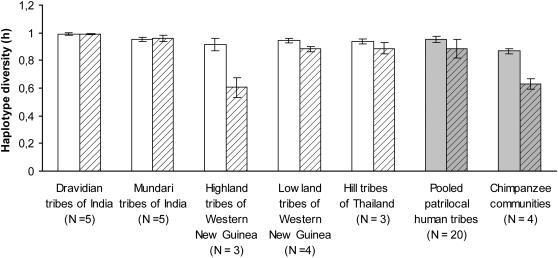
Mean and standard deviation for haplotype diversity of NRY (open bars) and mtDNA (thatched bars) for patrilocal human tribal groups and chimpanzees. For patrilocal humans, values for individual tribal groups (white bars) and the average of the pooled values of all 20 tribes (grey bars) are shown.

We found that the average NRY haplotype diversity of the four chimpanzee communities (h = 0.63, S.D. = 0.08) was significantly lower than that of the 20 patrilocal human tribes (h = 0.89, S.D. = 0.14) (Mann-Whitney U, *P* = 0.008, two-tailed) (Figure 1). However, the average level of mtDNA haplotype diversity was also significantly lower in the chimpanzees (h = 0.87, S.D. = 0.04) than in the patrilocal human tribes (h = 0.95, S.D. = 0.14) (Mann-Whitney U, *P* = 0.008, two-tailed) (Figure 1), suggesting that the lower diversity values in chimpanzees may simply reflect the low average levels of genetic variation typically observed in studies of the East African subspecies [20]. Nevertheless, the mean ratio of NRY/mtDNA haplotype diversity was significantly lower in chimpanzees (h = 0.73, S.D. = 0.08) than in patrilocal human tribes (h = 0.93, S.D. = 0.13) (Mann-Whitney U, *P* = 0.005, two-tailed). Chimpanzees thus have reduced levels of NRY diversity relative to mtDNA diversity compared with patrilocal humans.

**Figure 2:**
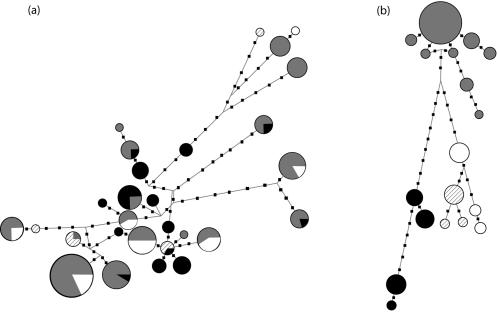
Median-joining networks of (a) mtDNA hypervariable region 1 sequences and (b) NRY microsatellites in the Ngogo (grey circles), Sonso (black circles), Kanyawara (white circles), and Mugiri (thatched circles) chimpanzee communities. Circle size is proportional to haplotype frequency. Small squares separating haplotypes represent mutations.

We found extensive sharing of mtDNA variants among chimpanzee communities that strongly contrasted with complete community specificity of NRY haplotypes (Figure 2). In contrast, both mtDNA and NRY haplotypes were shared between tribes within a patrilocal human tribal group in all of the five tribal groups (data not shown). As indicated by the lack of overlap in 95% confidence intervals, the average unstandardized NRY genetic differentiation among chimpanzee communities was significantly higher than among all of the patrilocal human tribal groups except the Western New Guinea Highlanders (Table 1). Unstandardized mtDNA genetic differentiation, however, was much more similar between chimpanzees and patrilocal humans (Table 1). Chimpanzees displayed the largest ratios of unstandardized NRY/mtDNA genetic differentiation. In chimpanzees and two of five patrilocal human tribal groups, i.e., Highland Western New Guinea and Thai Hill, unstandardized genetic differentiation was significantly larger for the NRY than the mtDNA.

**Table 1:** Unstandardized NRY and mtDNA genetic differentiation (with 95% confidence interval limits) and ratios of unstandardized NRY/mtDNA genetic differentiation in chimpanzees and patrilocal human tribal groups.

	NRY F_ST_	mtDNA F_ST_	NRY F_ST_/mtDNA F_ST_
Chimpanzee communities	0.83 (0.73–0.91)	0.07 (0.05–0.10)	11.9
Dravidian tribes of India	0.01 (0.00–0.06)	0.03 (0.02–0.04)	0.3
Mundari tribes of India	0.04 (0.01–0.07)	0.01 (0.00–0.03)	4.0
Highland tribes of Western New Guinea	0.57 (0.38–0.78)	0.20 (0.15–0.23)	2.9
Lowland tribes of Western New Guinea	0.16 (0.04–0.31)	0.03 (0.01–0.05)	5.3
Hill tribes of Thailand	0.39 (0.29–0.50)	0.08 (0.05–0.11)	4.9

Standardized genetic differentiation produced broadly similar results, with chimpanzees showing significantly larger standardized NRY genetic differentiation than three of five patrilocal human tribal groups (Table 2). However, compared to chimpanzees, standardized NRY genetic differentiation in patrilocal human tribal groups increased substantially relative to unstandardized genetic differentiation. Standardization did not appreciably alter mtDNA differentiation for either chimpanzees or patrilocal human tribal groups. Standardized NRY/mtDNA genetic differentiation ratios were thus larger than unstandardized ratios for patrilocal human tribal groups but not for chimpanzees. This resulted in a smaller difference between chimpanzees and patrilocal human tribal groups in standardized compared to unstandardized NRY/mtDNA genetic differentiation ratios.

**Table 2:** Standardized NRY and mtDNA genetic differentiation (with 95% confidence interval limits) and ratios of standardized NRY/mtDNA genetic differentiation in chimpanzees and patrilocal human tribal groups.

	NRY F_ST_	mtDNA F_ST_	NRY F_ST_/mtDNA F_ST_
Chimpanzee communities	0.97 (0.79–1.00)	0.09 (0.05–0.12)	10.8
Dravidian tribes of India	0.04 (0.00–0.22)	0.03 (0.02–0.04)	1.3
Mundari tribes of India	0.08 (0.02–0.21)	0.02 (0.00–0.03)	4.0
Highland tribes of Western New Guinea	0.77 (0.44–1.00)	0.22 (0.16–0.26)	3.5
Lowland tribes of Western New Guinea	0.30 (0.06–0.71)	0.04 (0.02–0.06)	7.5
Hill tribes of Thailand	0.72 (0.45–1.00)	0.08 (0.05–0.13)	9.0

**Figure 3:**
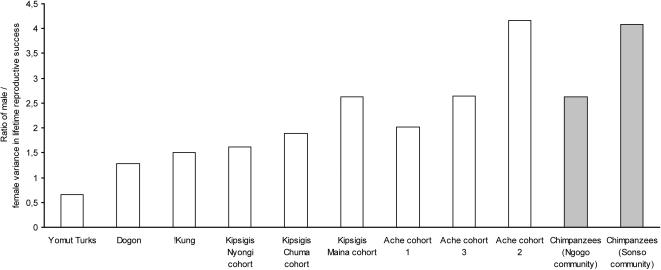
Ratio of male to female variance in lifetime reproductive success in traditional human societies (white bars) and chimpanzees (grey bars).

**Figure 4:**
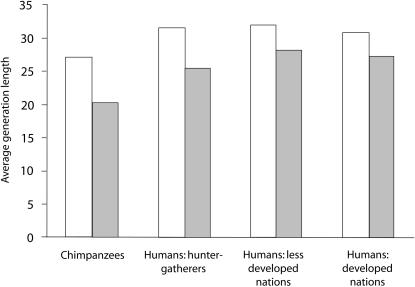
Average generation length of males (white bars) and females (grey bars) in chimpanzees and different types of human societies.

The more similar standardized than unstandardized NRY/mtDNA genetic differentiation ratios between chimpanzees and patrilocal human tribal groups suggest that in addition to differences in the extent of female-biased migration, other factors which reduce NRY relative to mtDNA within-group variation have affected chimpanzees more strongly than the patrilocal human tribal groups. One possibility is that the effective population size of males relative to females may be smaller in chimpanzees than it is in humans. To test this hypothesis, we used parentage assignments in the chimpanzee communities and published human data sets to compare sex differences in effective population size in both species. We found that the ratio of male to female variance in lifetime reproductive success (LRS), an important factor influencing sex differences in effective population size, is actually similar in chimpanzees and in humans living in traditional societies (Figure 3, see methods for an explanation for why the sex difference in variance in LRS for chimpanzees is actually likely to be even smaller than shown in this figure). Variations in generation length between the sexes can also cause differences in male and female effective population sizes. However, sex differences in average generation length are also similar for chimpanzees and humans (Figure 4). Taken together, these data indicate that the sex difference in effective population size is similar in chimpanzees and humans, and that another factor must explain why standardized NRY/mtDNA genetic differentiation ratios are more similar between chimpanzees and patrilocal human tribal groups compared to unstandardized ratios. One possibility is ascertainment bias. Unlike our assay of mtDNA variation, where both chimpanzees and humans were sequenced at comparable portions of the first hypervariable region of the mtDNA, we assessed chimpanzee NRY variation by typing microsatellite markers that were originally discovered as polymorphic in humans. This process may have led to an artificial downward bias in estimates of chimpanzee NRY variation, which would increase unstandardized NRY genetic differentiation in chimpanzees relative to patrilocal humans.

Whatever the reason for the discrepancy between unstandardized and standardized NRY/mtDNA genetic differentiation ratios, standardized NRY and mtDNA genetic differentiation were still more strongly contrasting in chimpanzees than in patrilocal human tribal groups. This suggests that migration is more female-biased between chimpanzee communities than between patrilocal human tribes within a tribal group. Additional analyses of patrilocal humans which are conducted on the scale over which sex-biased migration actually occurs, e.g., villages within a tribe rather than tribes within a tribal group, will be necessary to test this hypothesis. Alternatively, the recent history of patrilocality in most human populations may have prevented the build-up of such extremely contrasting patterns of mtDNA and NRY variation. For humans, the absence of higher NRY than mtDNA genetic differentiation at the continental and global scale may indicate that migration is not female-biased at these broad geographical distances [6]. Yet another possibility suggested by a recent simulation study is that worldwide migration is in fact currently female-biased, but that there has not been enough time for this migration pattern to erase the bilocal migration pattern that existed for the majority of humanity's evolutionary history as foragers [21]. Comparisons of patterns of mtDNA and NRY variation at the local and broader geographic scales in chimpanzees, which have a long evolutionary history of female-biased migration, could provide a complementary empirical test of this hypothesis.

## Materials and Methods

### Population sampling and laboratory methods

We examined mtDNA and NRY variation in four communities of chimpanzees (number of mtDNA/NRY-chromosomes sampled in parentheses): the Sonso (28/16) community of Budongo Forest Reserve, the Ngogo (94/41) and Kanyawara (20/10) communities of Kibale National Park, and the Mugiri (7/6) community of Semliki National Park. We also analyzed previously published data from five patrilocal Dravidian tribes from India (Vanne (32/23), Pokanati (59/25), Panta (37/21), Kapu (22/16) and Akhutota (32/21)), five patrilocal Mundari tribes from India (Santhal (39/38), Munda (23/23), Kharia (21/13), Bhumij (40/39) and Asur (30/28)), three patrilocal Hill tribes from Thailand (Akha (91/21), Chiang Rae Lisu (53/9) and Mae Hong Son Lisu (42/22)), four patrilocal tribes from the lowlands of Western New Guinea (Awyu (12/10), Citak (40/28), Mappi (18/10) and Muyu (10/6)), and three patrilocal tribes from the highlands of Western New Guinea (Dani (21/12), Ketengeban (22/19) and Una (51/46). Subjects were sequenced at the first hypervariable control region of the mtDNA and genotyped at microsatellite loci on the NRY. The chimpanzees were sequenced at 473 bases and genotyped at 9 microsatellite loci following procedures described elsewhere [13], [22]. The Genbank (http://www.ncbi.nlm.nih.gov) accession numbers for the chimpanzee mtDNA sequences are EU077270-EU077418. Supplementary Table S1 shows the chimpanzee NRY microsatellite haplotypes and their occurrences in the four communities. The Dravidian and Mundari tribes were sequenced at up to 350 mtDNA bases and 6 NRY microsatellite loci, the Thai Hill tribes at up to 360 bases and 9 loci, and the Western New Guinea tribes at up to 350 bases and 7 loci, as described in the original publications [3], [5], [17].

### Analytical procedures

We used Arlequin 3.10 [23] to estimate haplotype diversity [24] for mtDNA and NRY within groups and genetic differentiation between groups. We conducted Mann-Whitney *U* tests to compare average mtDNA haplotype diversity, NRY haplotype diversity, and the ratio of NRY/mtDNA haplotype diversity between the chimpanzee communities (*N* = 4) and the patrilocal human tribes (*N* = 20).

Unstandardized genetic differentiation was calculated in an AMOVA framework, with the number of different alleles used as the distance metric for both mtDNA and NRY. We examined the overlap in the 95% confidence limits generated by bootstrapping genetic differentiation values in a locus-by-locus AMOVA to evaluate whether (a) genetic differentiation between the chimpanzees and each of the five patrilocal human tribal groups were significantly different and (b) genetic differentiation of NRY and mtDNA were significantly different within chimpanzees and patrilocal human tribal groups.

To calculate standardized genetic differentiation, we first transformed the data such that each population had its own set of unique alleles (e.g., when there were two populations with alleles *a* and *b* at a locus, in one of the populations these alleles were recoded as *c* and *d*), and calculated an AMOVA F_ST_ for this transformed data set (mtDNA/NRY chimpanzees: 0.85/0.86; Dravidian: 0.93/0.33; Mundari 0.93/0.49; Western New Guinea highland: 0.88/0.75; Western New Guinea lowland: 0.86/0.53; Thai Hill: 0.91/0.55). This transformation does not affect within-population variation, but maximizes between-population variation. We then calculated standardized genetic differentiation by dividing the original unstandardized AMOVA F_ST_ by the AMOVA F_ST_ obtained for this transformed dataset. We included only variable loci when calculating both unstandardized and standardized genetic differentiation. The inclusion or exclusion of non-variable loci does not affect the unstandardized AMOVA, but including non-variable loci when calculating the AMOVA for the transformed data set results in AMOVA values that are determined more by the ratio of variable to non-variable sites, and less by the levels of variability of the variable loci. This would push maximum genetic differentiation possible towards 1, and result in standardized genetic differentiation values that are nearly identical to their unstandardized counterparts. To determine 95% confidence intervals for the standardized genetic differentiation, we divided the lower 95% confidence limit of the original unstandardized genetic differentiation value by the upper 95% confidence limit of the transformed genetic differentiation, and divided the upper 95% confidence limit of the original unstandardized genetic differentiation by the lower 95% confidence limit of the transformed genetic differentiation. For all tests, loci had to have less than 5% missing data to be included in calculations.

To estimate variance in lifetime reproductive success, we calculated the variance in the number of offspring divided by the square of the mean number of offspring, for both males (I_M_) and females (I_F_). I_M _and I_F _are commonly used to estimate variance in lifetime reproductive success when the sampling of male and female reproductive output is incomplete and the data indicate the sexes have different mean lifetime reproductive success, when in reality they must be equal [25]. For traditional human societies, mean and variance in offspring numbers were collected from published data sets (Yomut Turks: [26]; Dogon: [27]; !Kung: [28]; Kipsigis: [29]; Ache: [30]). The data sets differed in how offspring number was measured, e.g., number of offspring produced in a lifetime versus number of offspring currently living, and in other methodological details, as described in the original publications. For the chimpanzees, we conducted maternity and paternity analyses of the Sonso and Ngogo communities using 9–44 autosomal microsatellite loci. Our sample included 26 adult females and 18 adult males from the Sonso community, and 41 adult females and 27 adult males from the Ngogo community. As very few of the chimpanzees had completed their reproductive careers, our estimates reflect short-term variance in reproductive success rather than true variance in LRS. Studies of primates and other mammals typically show that for females, short-term estimates of variance in reproductive success underestimate lifetime variance in reproductive success [25], [31]. This occurs because longevity is a key component of variance in LRS for females [25], [31]. In contrast, short-term estimates of variance in reproductive success for males typically overestimate variance in LRS, as most of a male's reproduction occurs during a narrow period of life when he can successfully compete with other males for fertilizations [32]. Although estimates of variance in LRS are currently unavailable for male chimpanzees, long-term observations from the Mahale chimpanzee study site indicate that variance in LRS for females is approximately twice as large as our short-term estimates from Sonso and Ngogo [33]. Thus, our estimates for chimpanzees are likely to substantially overestimate the extent to which variance in male LRS exceeds that of females.

We calculated the average ages of mothers and fathers at the time of birth of 19 Sonso offspring to compute female and male average generation lengths in chimpanzees. We did not include the Ngogo data, as ages of adult males and females are less well-known in this community. Human values are from [34].

## Supporting Information

Table S1 NRY microsatellite haplotypes and their occurrences in four chimpanzee communities.
(0.03 MB DOC)

## References

[1] Murdoch GP (1981). Atlas of World Cultures..

[2] Fox RF (1967). Kinship and Marriage..

[3] Oota H, Settheetham-Ishida W, Tiwawech D, Ishida T, Stoneking M (2001). Human mtDNA and Y-chromosome variation is correlated with matrilocal versus patrilocal residence.. Nat Genet.

[4] Chaix R, Quintana-Murci L, Hegay T, Hammer MF, Mobasher Z (2007). From social to genetic structures in central Asia.. Curr Biol.

[5] Kumar V, Langstieh BT, Madhavi KV, Naidu VM, Singh HP (2006). Global patterns in human mitochondrial DNA and Y-chromosome variation caused by spatial instability of the local cultural processes.. PLoS Genet.

[6] Wilder JA, Kingan SB, Mobasher Z, Metni Pilkington M, Hammer MF (2004). Global patterns of human mitochondrial DNA and Y-chromosome structure are not influenced by higher migration rates of females versus males.. Nat Genet.

[7] Seielstad MT, Minch E, Cavalli-Sforza LL (1998). Genetic evidence for a higher female migration rate in humans.. Nat Genet.

[8] Wilder JA, Mobasher Z, Hammer MF (2004). Genetic evidence for unequal effective population sizes of human males and females.. Mol Biol Evol.

[9] Alvarez HP, Chapais B, Berman CM (2004). Residence groups among hunter-gatherers.. Kinship and Behavior in Primates.

[10] Whitlock MC, McCauley DE (1999). Indirect measures of gene flow and migration: F_ST_≠1(4*Nm* = 1).. Hered.

[11] Kano T (1992). The Last Ape..

[12] Swedell L, Sussman RW, Vasey N (2006). Strategies of Sex and Survival in Hamadryas Baboons: Through a Female Lens;.

[13] Eriksson J, Siedel H, Lukas D, Kayser M, Erler A (2006). Y-chromosome analysis confirms highly sex-biased dispersal and suggests a low male effective population size in bonobos (*Pan paniscus*).. Mol Ecol.

[14] Hammond RL, Lawson Handley LJ, Winney BJ, Bruford MW, Perrin N (2005). Genetic evidence for female-biased dispersal in a polygynous primate.. P Roy Soc Lond B Bio.

[15] Mitani JC, Watts DP, Muller MN (2002). Recent developments in the study of wild chimpanzee behavior.. Evol Anthropol.

[16] Won Y-J, Hey J (2005). Divergence population genetics of chimpanzees.. Mol Biol Evol.

[17] Kayser M, Brauer S, Weiss G, Schiefenhovel W, Underhill P (2003). Reduced Y-chromosome, but not mitochondrial DNA, diversity in human populations from West New Guinea.. Am J Hum Genet.

[18] Hedrick PW (2005). A standardized genetic differentiation measure.. Evol.

[19] Meirmans PG (2006). Using the AMOVA framework to estimate a stndardized genetic differentiation measure.. Evol.

[20] Gagneux P, Gonder MK, Goldberg TL, Morin PA (2001). Gene flow in wild chimpanzee populations: what genetic data tell us about chimpanzee movement over space and time.. P Roy Soc Lond B Bio.

[21] Wilkins JF, Marlowe FW (2005). Sex-biased migration in humans: what should we expect from genetic data?. BioEssays.

[22] Eriksson J, Hohmann G, Boesch C, Vigilant L (2004). Rivers influence the population genetic structure of bonobos (*Pan paniscus*).. Mol Ecol.

[23] Excoffier L, Laval G, Schneider S (2005). Arlequin ver. 3.0: An integrated software package for popuation genetics data analysis.. Evol Bioinf Online.

[24] Nei M (1987). Molecular Evolutionary Genetics..

[25] Clutton-Brock T (1988). Reproductive Success..

[26] Irons W, Cronk L, Chagnon N, Irons W (2000). Why do the Yomut raise more sons than daughters.. Adaptation and human behavior: an evolutionary prespective.

[27] Strassmann BI, Reichard UH, Boesch C (2003). Social monogamy in a human society: marriage and reproductive success among the Dogon.. Monogamy: Mating strategies and partnerships in birds, humans and other animals.

[28] Howell N (1979). Demography of the Dobe !Kung..

[29] Borgerhoff Mulder M, Clutton-Brock TH (1988). Reproductive success in three Kipsigis cohorts.. Reproductive Success.

[30] Hill K, Hurtado AM (1996). Ache Life History..

[31] Rhine RJ, Norton GW, Wasser SK (2000). Lifetime reproductive success, longevity, and reproductive life history of female yellow baboons (*Papio cynocephalus*) of Mikumi National Park, Tanzania.. Am J Primatol.

[32] Altmann J, Alberts SC, Haines SA, Dubach J, Muruthi P (1996). Behavior predicts genetic structure in a wild primate group.. Proc Natl Acad Sciences USA.

[33] Nishida T, Corp N, Hamai M, Hasegawa T, Hiraiwa-Hasegawa M (2003). Demography, female life history, and reproductive profiles among the chimpanzees of Mahale.. Am J Primatol.

[34] Fenner JN (2005). Cross-cultural estimation of the human generation interval for use in genetics-based population divergence studies.. Am J Phys Anthropol.

