# Supplementary material for: The Genetic Signature of Sex-Biased Migration in Patrilocal Chimpanzees and Humans
Source: PLoS One. 2007 Oct 3;2(10):e973. doi: 10.1371/journal.pone.0000973 (PMC1989134; doi:10.1371/journal.pone.0000973)
Supplement: Table S1 — NRY microsatellite haplotypes and their occurrences in four chimpanzee communities. (0.03 MB DOC) [file pone.0000973.s001.doc]

Table S1. NRY microsatellite haplotypes and their occurrences in four chimpanzee communities.

| DYS502 | DYS520 | DYS533 | DYS510 | DYS517 | DYS612 | DYS630 | DYS469 | DYS588 | Kanyawara | Ngogo | Sonso | Mugiri |
| --- | --- | --- | --- | --- | --- | --- | --- | --- | --- | --- | --- | --- |
| 247 | 150 | 210 | 234 | 200 | 202 | 161 | 243 | 174 | 0 | 27 | 0 | 0 |
| 247 | 150 | 210 | 234 | 200 | 202 | 161 | 243 | 179 | 0 | 4 | 0 | 0 |
| 247 | 150 | 214 | 234 | 200 | 202 | 161 | 243 | 174 | 0 | 2 | 0 | 0 |
| 247 | 150 | 210 | 234 | 200 | 196 | 161 | 240 | 174 | 0 | 3 | 0 | 0 |
| 247 | 150 | 206 | 234 | 200 | 202 | 161 | 243 | 179 | 0 | 2 | 0 | 0 |
| 247 | 150 | 210 | 234 | 200 | 205 | 161 | 243 | 174 | 0 | 1 | 0 | 0 |
| 247 | 150 | 210 | 234 | 200 | 193 | 161 | 243 | 174 | 0 | 1 | 0 | 0 |
| 247 | 150 | 210 | 234 | 200 | 202 | 161 | 240 | 174 | 0 | 1 | 0 | 0 |
| 243 | 142 | 206 | 238 | 200 | 205 | 165 | 237 | 174 | 6 | 0 | 0 | 0 |
| 243 | 142 | 206 | 238 | 200 | 199 | 169 | 237 | 179 | 2 | 0 | 0 | 0 |
| 243 | 142 | 206 | 234 | 200 | 199 | 169 | 237 | 179 | 2 | 0 | 0 | 0 |
| 243 | 142 | 206 | 238 | 200 | 205 | 177 | 237 | 174 | 0 | 0 | 0 | 4 |
| 243 | 142 | 206 | 238 | 200 | 205 | 181 | 234 | 174 | 0 | 0 | 0 | 1 |
| 243 | 142 | 206 | 238 | 204 | 205 | 177 | 237 | 169 | 0 | 0 | 0 | 1 |
| 235 | 158 | 206 | 230 | 204 | 199 | 165 | 231 | 184 | 0 | 0 | 6 | 0 |
| 247 | 158 | 210 | 230 | 204 | 205 | 165 | 231 | 184 | 0 | 0 | 4 | 0 |
| 247 | 158 | 210 | 230 | 204 | 208 | 165 | 231 | 184 | 0 | 0 | 5 | 0 |
| 235 | 158 | 206 | 230 | 208 | 199 | 165 | 231 | 184 | 0 | 0 | 1 | 0 |
